# Soft Magnetic Powdery Sensor for Tactile Sensing

**DOI:** 10.3390/s19122677

**Published:** 2019-06-13

**Authors:** Shunsuke Nagahama, Kayo Migita, Shigeki Sugano

**Affiliations:** 1 Waseda Research Institute for Science and Engineering, Waseda University, 3-4-1 Okubo, Shinjuku-ku, Tokyo 169-8555, Japan; 2 School of Creative Science and Engineering, Waseda University, 3-4-1 Okubo, Shinjuku-ku, Tokyo 169-8555, Japan; k-migita@moegi.waseda.jp (K.M.); sugano@waseda.jp (S.S.)

**Keywords:** ferromagnetic powder, soft material, tactile sensor, magnetic field orientation

## Abstract

Soft resistive tactile sensors are versatile devices with applications in next-generation flexible electronics. We developed a novel type of soft resistive tactile sensor called a soft magnetic powdery sensor (soft-MPS) and evaluated its response characteristics. The soft-MPS comprises ferromagnetic powder that is immobilized in a liquid resin such as polydimethylsiloxane (PDMS) after orienting in a magnetic field. On applying an external force to the sensor, the relative distance between particles changes, thereby affecting its resistance. Since the ferromagnetic powders are in contact from the initial state, they have the ability to detect small contact forces compared to conventional resistive sensors in which the conductive powder is dispersed in a flexible material. The sensor unit can be made in any shape by controlling the layout of the magnetic field. Soft-MPSs with different hardnesses that could detect small forces were fabricated. The soft-MPS could be applied to detect collisions in robot hands/arms or in ultra-sensitive touchscreen devices.

## 1. Introduction

Tactile sensors are important components in robots as they enable them to detect objects. In recent years, soft sensors have been developed for a multitude of applications, with the aim of enhancing interactions between human beings and machines. There are various types of sensors such as magnetic, piezoelectric, resistive, capacitive, and optical [1,2,3]. Research is also being conducted on technology that can estimate the force of an end effector or load on a joint without using a force sensor [4,5,6]. Capacitive touch sensors measure applied force from changes in their capacitance [7,8,9,10,11]. In these sensors, two sets of electrodes are placed opposite each other with a dielectric layer sandwiched in between. On applying a force, the distance between the electrodes changes; the resulting change in electrostatic capacity is a measure of the force applied. Due to the nature of the detection principle, these capacitive sensors are susceptible to environmental noise. In contrast, magnetic tactile sensors measure applied force from changes in the magnetic field [12,13,14,15]. In this method, displacement and force are estimated by measuring the change in magnetic flux density caused by the change in position of the magnetic particles embedded in the material. This is carried out by measuring the magnetic Hall voltage and the voltage change generated by self-electromotive force on the inducer. The main drawback of detecting changes in magnetic properties is that they are susceptible to the effects of geomagnetism and stray fields caused by magnetic materials in the environment. Optical tactile sensors, in contrast, measure displacement or force by observing the optical properties due to deformation of the material. Changes in light reflection due to the change in dot-patterns or deformation are observed using charge-coupled device (CCD) cameras [16,17,18,19]. However, since it is necessary to observe such changes optically, a certain distance is required between the material and the sensor, imposing restrictions on the size and geometry of the sensor. Piezoelectric tactile sensors measure applied force by evaluating the voltage generated when pressure is applied to a piezoelectric material [20,21]. However, since the piezoelectric effect arises from distortion of the crystal symmetry, it is difficult to construct flexible sensors out of these materials.

In this study, we focused on resistive sensors that are less susceptible to disturbances. Resistive tactile sensors include liquid type sensors, strain gauge sensors, and sensors using conductive threads [22,23,24]. Resistance-type sensors have also been fabricated from conductive powder dispersed in soft material such as Inastomer [25,26]. In this method, when the amount of conductive powder is small, conducting channels are created only upon the application of pressure; when no pressure is applied, no conduction occurs. Hence, the sensitivity is poor. However, the conductive powder is often a hard substance such as metal or carbon, so as its concentration increases, the flexibility of the sensor is lost. Magnetic compound fluid (MCF) rubber sensors are fabricated by confining a fluid in a magnetic field, which is then crosslinked by heat or electrolytic polymerization [27,28,29,30]. The hardness of this sensor depends on the hardness after polymerization.

We previously developed a type of tactile sensor that uses ferromagnetic powder as a sensing element [31]. In these “magnetic powdery sensors” (MPSs), ferromagnetic powder is confined within magnetic field lines from permanent magnets mounted on parallel plates. As the distance between the plates decreases, the spatial aggregation pattern of the ferromagnetic powder changes, which is reflected in the electrical resistance. Hence, the displacement can be measured by observing the change in resistance. One of the distinguishing features of this measurement technique is its linearity. However, since the powder is merely trapped in the magnetic field, it can be displaced upon vibration or impact. Furthermore, a mechanism such as a spring is required to restore the initial distance between the plates, which increases the size of the device.

Based on these methodologies, we devised a sensor comprising a small amount of conductive powder embedded in a soft gel matrix, wherein the powder is confined using a magnetic field to form conductive channels. Unlike in MPSs, where the powder is held in place only by the magnetic field, in these novel sensors, once the powder is trapped within the magnetic field lines, it is held in place with a silicone gel to prevent displacement. Moreover, the elasticity of the silicone rubber is used as a restoring force, negating the need for a spring-type mechanism. By utilizing this technique with a suitable powder and soft material, a highly sensitive and flexible tactile sensor can be realized. Hereafter, this sensor is referred to as a soft-MPS.

## 2. Materials and Methods

**Figure 1:**
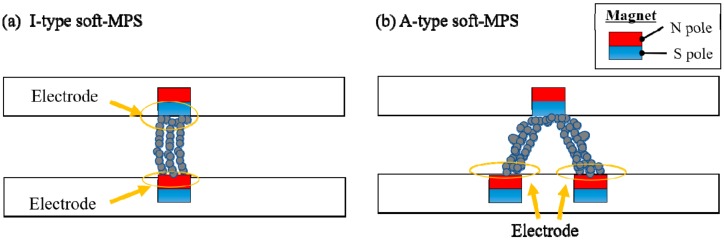
Magnetic field orientation of the ferromagnetic powder for two different types of magnetic resistive tactile sensors: (**a**) I-type soft-magnetic powdery sensors (MPS). One of the electrodes is set on the plate to which the external force is applied. (**b**) A-type soft-MPS. All electrodes are set on the bottom side.

In a magnetic tactile sensor, the magnetic field can be oriented in different ways. General resistive sensors typically use the arrangement shown in Figure 1a. However, in this arrangement, it is necessary to place an electrode on the surface to which force is applied, which reduces the durability of the sensor. In the proposed soft-MPS, the electrodes are placed on the bottom plate alone by devising an arrangement as shown in Figure 1b.

**Figure 2:**
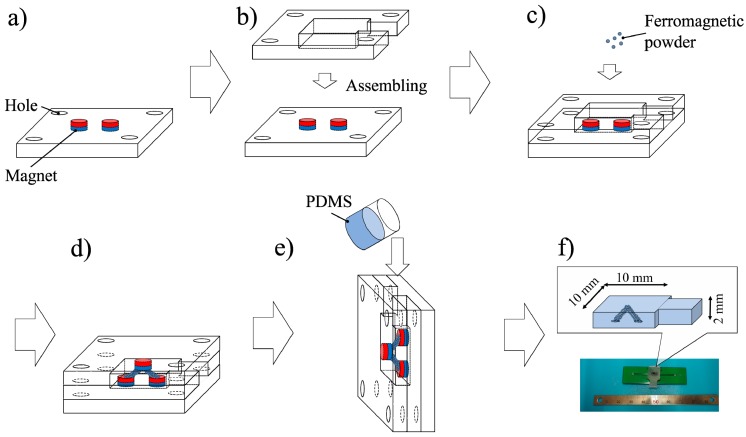
Fabrication process of the soft-MPS: (**a**) magnets (⌀1 × 5 mm, 198 mT) are installed inside a 3D printed mold; (**b**) a spacer (thickness: 2 mm) is set for pouring the polydimethylsiloxane (PDMS) solution; (**c**) ferromagnetic powder (200 mesh) is added into the mold with the magnets; (**d**) the top lid is attached to bridge the ferromagnetic powder; (**e**) PDMS is poured into the mold; (**f**) the soft-MPS (area: 10 mm^2^, thickness: 2 mm) is obtained and the sensor is fixed on a printed circuit board with a conductive adhesive.

Figure 2 schematically shows the manufacturing process of the soft-MPS. The soft-MPS consists of a case made of acrylonitrile butadiene styrene (ABS) and a 3D-printed magnet. First, the sensor unit was constructed by arranging iron powder along the field lines produced by a magnetic field. Following this, a sensor sheet was manufactured by fixing this iron powder in a matrix of silicone rubber. The electrode was subsequently fixed on the top of the sensor sheet using a silicone primer (PPX primer, CEMEDINE Co. Ltd., Tokyo, Japan) and a silver nano-adhesive. The primer improves the adhesion between the silicone and silver adhesive. This arrangement was left standing at room temperature for 24 h, and the soft-MPS was thus fabricated.

**Figure 3:**
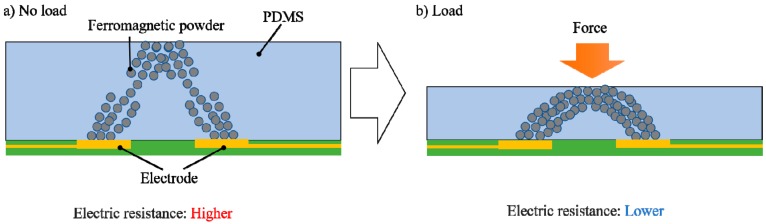
The principle of resistance measurement on soft-MPS for: (**a**) initial condition; (**b**) load condition.

Figure 3 shows the resistance measurement principle for the soft-MPS. On applying an external force, the intergranular distance of the ferromagnetic powder changes. As a result, the resistance changes, and the displacement as well as the force can be measured. By trapping the ferromagnetic powder in a magnetic field during the fabrication process, it becomes conductive even with a small amount of metal. Therefore, unlike a pressure-sensitive material such as Inastomer (developed by Inaba Rubber Co. Ltd., Osaka, Japan), in which a conductive substance such as carbon is dispersed in rubber, the soft-MPS has a finite electrical resistance even in the absence of an external force. When a force is applied, the ferromagnetic powder that has been oriented in the magnetic field changes its position. It is hypothesized that if the ferromagnetic powder is aligned along the magnetic field, the change in resistance due to the applied force will be small. In previous MPS experiments, the change in the resistance was linear with the change in the amount of ferromagnetic powder trapped by the magnetic field lines [31]. On the contrary, in the sensor used here, the powder is oriented by the magnetic field only at the time of manufacturing. After fabrication, the resistance changes due to the change in the positional relationship of the powder, since there is no confinement by the magnetic field. Moreover, the MPS measures the changes in resistance over a relatively large gap (about 0 to 30 mm). Since there are not many real-world scenarios where such a force is applied to a tactile sensor, it is necessary to investigate the characteristics for smaller areas and displacements, which is the case for the soft-MPS used in this study.

## 3. Experiments and Results

**Figure 4:**
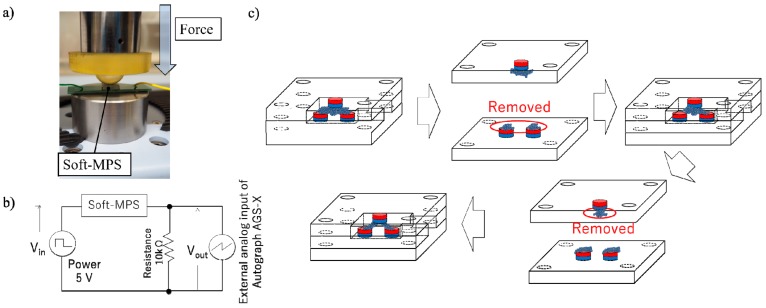
The experimental setup: (**a**) loading of the soft-MPS; (**b**) circuit to detect the voltage change. The voltage was measured at the resistor (10 kΩ); (**c**) removing excess iron powder.

We measured the change of voltage with respect to the force applied to the soft-MPS. The resistance of the soft-MPS changes upon applying an external force, and as a result, the voltage through the soft-MPS changes. A jig capable of pressing a hemisphere of diameter 10 mm on the sensor was attached to a universal testing machine (Autograph AGS-X, Shimadzu Corporation, Kyoto, Japan), and the applied force and change in voltage were measured as a result of the displacement upon pressing the sensor (Figure 4b). Since the pressure was measured, the sensitivity changes upon changing the value of resistance (R). In this study, we measured in advance the change in voltage of the sensor when R was changed, and adopted 10 kΩ for all subsequent measurements since the output of the sensor was stable at that value. The amount of ferromagnetic powder (200 mesh iron powder, Kyowa Pure Chemical Co. Ltd., Tokyo, Japan) in the soft-MPS was adjusted as shown in Figure 4c. All experiments were conducted at room temperature (23 °C).

For the soft rubber matrix, polydimethylsiloxane (PDMS) (Ecoflex 00-50, Smooth-On, Inc., Macungie, PA, USA) and Sylgard 184 (Dow Corning Corporation, Midland, MI, USA) were used. The soft-MPS using Ecoflex 00-50 has lower hardness than the one using Sylgard 184. Hence, the soft-MPS using Sylgard 184 is considered capable of measuring relatively larger loads. In this experiment, the characteristics of soft-MPSs with different hardnesses were evaluated. Using the circuit shown in Figure 4a, the change in voltage of the soft-MPS upon external pressure was measured. The sensor manufactured using Ecoflex 00-50 is hereinafter referred to as soft-MPS (soft) and the sensor manufactured using Sylgard 184 as soft-MPS (hard).

### 3.1. Evaluation of Hysteresis

Changes in force and voltage were measured using Autograph at 1 mm/min giving a reciprocating displacement of 1 mm to the soft-MPS five times, and its hysteresis characteristics were measured.

**Figure 5:**
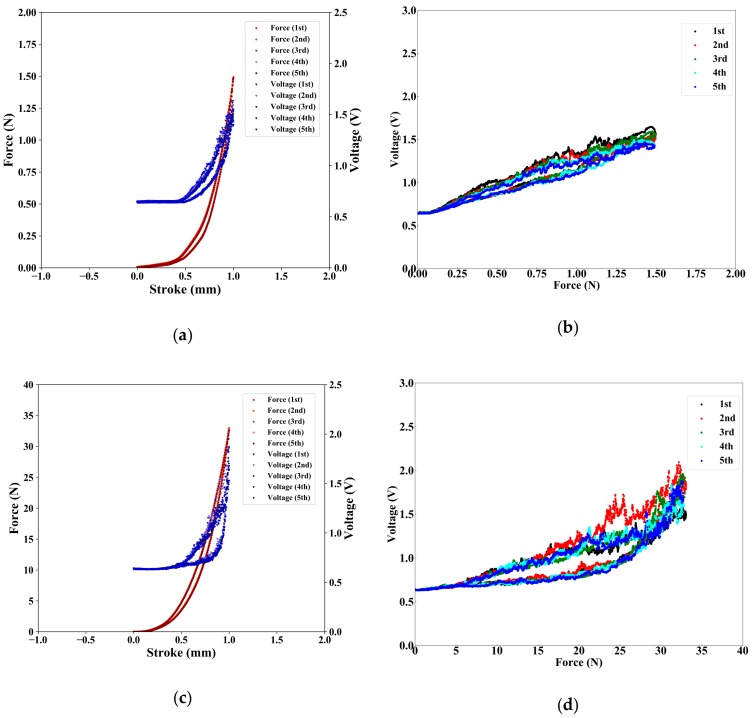
Results of evaluation of hysteresis. (**a**) Changes of force (from a reference value) and voltage in soft-MPS (soft) vs. stroke (1 mm/s). (**b**) Change of voltage in soft-MPS (soft) vs. force. (**c**) Changes of force and voltage in soft-MPS (hard) vs. stroke (1 mm/s). (**d**) Change of voltage in soft-MPS (hard) vs. force.

The experimental results are shown in Figure 5. Figure 5a,c show the relation between the force and the voltage in soft-MPS (soft) and soft-MPS (hard), respectively. Figure 5b,d show the relationship between the force and the voltage with respect to the number of the displacement of the soft-MPS (soft) and soft-MPS (hard), respectively.

From the fact that the voltage dropped as the resistance increased, it can be seen that the resistance of both sensors decreased as force increased. The larger range of force measured by soft-MPS (hard) than soft-MPS (soft) for the same displacement was due to its larger storage modulus. Furthermore, focusing on the area where the force was applied in soft-MPS (soft), the voltage started to decrease from a small force. On the contrary, the voltage of soft-MPS (hard) started to drop from 1 N.

### 3.2. Evaluation of Response for Controlled Force-Input

**Figure 6:**
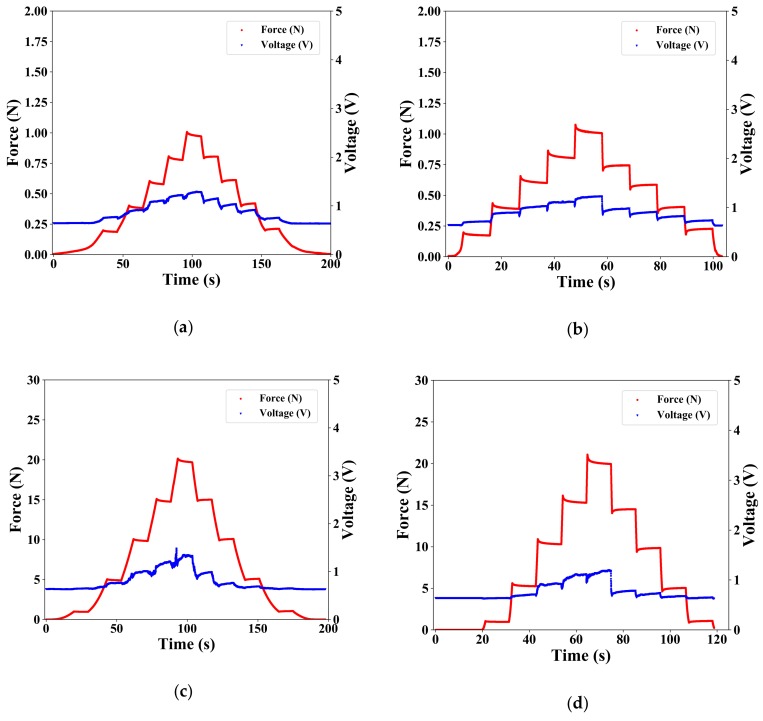
Results of the response for force-maintained input: (**a**,**b**) soft-MPS (soft) and (**c**,**d**) soft-MPS (hard) at testing rates of (**a**,**c**) 1 mm/s and (**b**,**d**) 10 mm/s.

Using Autograph, we measured the response of the soft-MPSs when applying a constant force for a fixed period of time. For soft-MPS (soft), forces of 0.2, 0.4, 0.6, 0.8, and 1.0 N were applied and then unloaded in stages. For soft-MPS (hard), forces of 1.0, 5.0, 10, 15, and 20 N were applied and then unloaded in stages. Figure 6a,c show the changes in force and voltage when force was applied to soft-MPS (soft) and soft-MPS (hard), respectively, at a displacement rate of 1 mm/min. Figure 6b,d show the corresponding trends for the soft-MPS (soft) and soft-MPS (hard), respectively, when displaced at 10 mm/min.

For soft-MPS (soft), it can be seen that the output changed in a step-like manner in response to the applied force. For soft-MPS (hard), the response when applying a force of 5 N was small. However, when applying a force of 10 N or more, the output voltage changed in a step-like manner. In addition, when the force was unloaded from either sensor, a large change in voltage was observed compared to when the force was applied.

### 3.3. Evaluation of Response for a Pulsed Force

**Figure 7:**
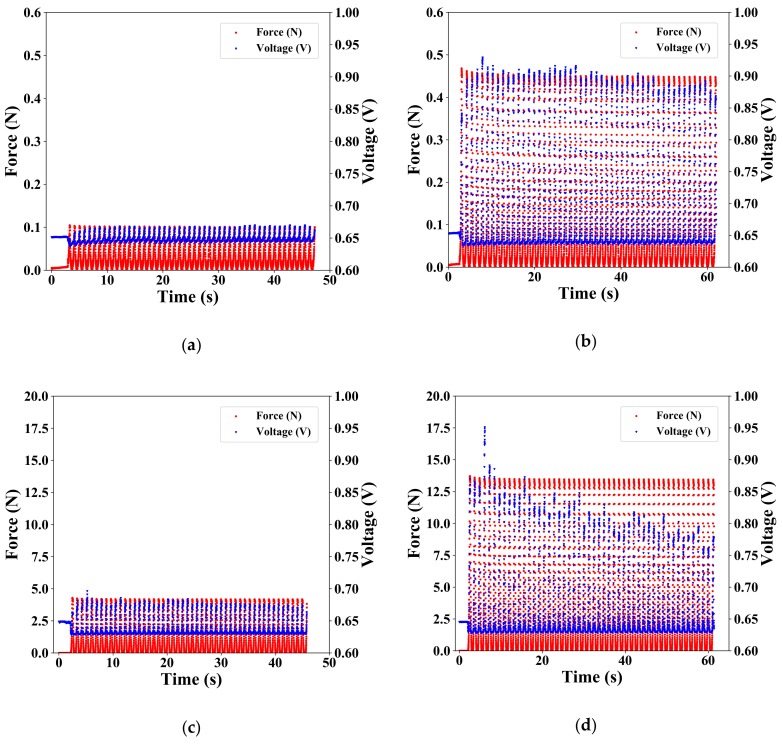
Results of the response for pulsed input: (**a**,**b**) soft-MPS (soft) and (**c**,**d**) soft-MPS (hard) at a pushing depth of (**a**,**c**) 0.5 mm and (**b**,**d**) 0.75 mm.

Using Autograph, changes in force and voltage were measured when a constant displacement was repeatedly applied to the soft-MPSs 50 times at 100 mm/min. Figure 7a,b show the voltage response of soft-MPS (soft) at a given displacement of 0.5 and 0.75 mm, respectively. Figure 7c,d show the voltage response of soft-MPS (hard) at a given displacement of 0.5 and 0.75 mm, respectively. From each graph, it can be seen that the voltage changed following the application of pressure in either sensor. In addition, it can be seen that the smaller the displacement, the smaller the scatter as the number of presses increased. Furthermore, from the fact that the peak of the sensor’s response voltage coincided with the peak of the applied force in time, it can be concluded that the sensor showed quick response to the applied force.

## 4. Discussion

### 4.1. Sensitivity of Soft-MPS

From Figure 5b,d, the sensitivities of the sensors can be determined as 560 mV/N for soft-MPS (soft) and 35 mV/N for soft-MPS (hard). These values largely correspond to the responses in Figure 6. In terms of sensitivity, it shows the same performance as other micro-electro-mechanical systems (MEMS) sensors [1,2,3].

**Figure 8:**
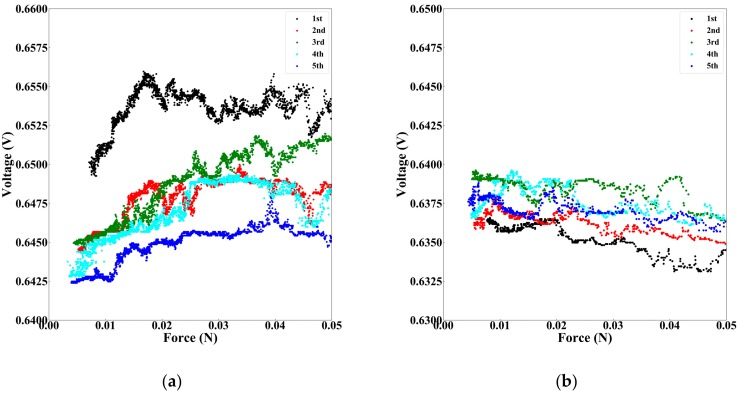
The response of soft-MPS in low load area. (**a**) Changes of voltage on soft-MPS (soft) vs. force. (**b**) Change of voltage on soft-MPS (hard) vs. force.

Figure 8 presents an enlarged view of the low force region of the response voltage against applied force (Figure 5b,d). As shown in Figure 8a, the voltage of soft-MPS (soft) had an overall tendency to increase with force, and it was possible to detect small changes in force. The third cycle had the most straightforward change in resistance to force. The output decreased as the number of cycles increased. In contrast, as shown in Figure 8b, the voltage of soft-MPS (hard) showed a slight tendency to decrease with increasing force. This difference is caused by the difference in hardness of the material. Soft-MPS (soft), which is composed of Ecoflex 00-50, is relatively softer than soft-MPS (hard), which is composed of Sylgard 184. It is believed that soft-MPS (soft) has higher detection sensitivity since it is easier to deform than soft-MPS (hard). One of the reasons why the output value of the sensors varied depending on the number of trials is that the sensor does not completely return to the original position when the force is unloaded. In addition, even though the powder is fixed inside a soft material, there is a possibility that the voltage may drop off slightly when a force is applied.

### 4.2. Response for a Pulsed Force

**Figure 9:**
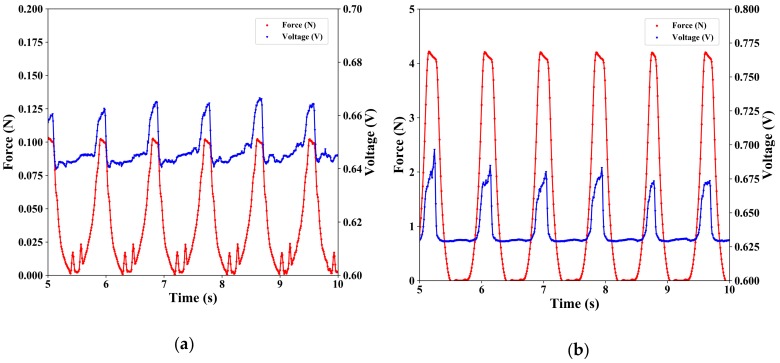
Response for pulsed input with a pushing depth of 0.5 mm: (**a**) soft-MPS (soft); (**b**) soft-MPS (hard).

The response when a pulsed input was applied (Figure 7) appeared at first to have no delay. A closer look, however, shows that there was a time lag between the applied force and the peak of the voltage response (Figure 9). It can be seen from the graph that the time delay for the response is about 40–100 ms. The same tendency was observed in both soft-MPSs. The powder in the sensing part of the soft-MPS moves with the deformation of the flexible material (PDMS), resulting in a time delay until the internal stress changes after the force is transmitted. This is an unavoidable situation when a soft material is used.

From the response time, we estimate the response rate as 10–15 Hz. This value is not particularly good compared to other sensors [1,2,3] or human skin sensors. However, it is considered that the long time to transmit the force could be related to the large size of the sensor unit. We will therefore investigate whether the transmission speed can be increased by making the sensor thinner and smaller.

### 4.3. Model of Soft-MPS

Modeling the resistance change in the soft-MPS upon the application of force is complex. In its simplest form, the mechanism of sensing depends on the fact that the compressive force changes depending on the buckling deflection based on the theory of general buckling of rectangular plates [32]. Nevertheless, in the real world, there are other mechanical forces generated from the deformation of the materials, as well as thermal forces [33]. Hence, the function of the soft-MPS is affected by a variety of parameters, and the change in resistance is caused by numerous factors in addition to strain and compressive force. Moreover, it would be necessary to estimate the mode of aggregation of the metal powder in the magnetic field as well as to analyze how the particles move in PDMS. Complete modeling of the sensor will be carried out in the future.

### 4.4. Arrayed Soft-MPS

**Figure 10:**
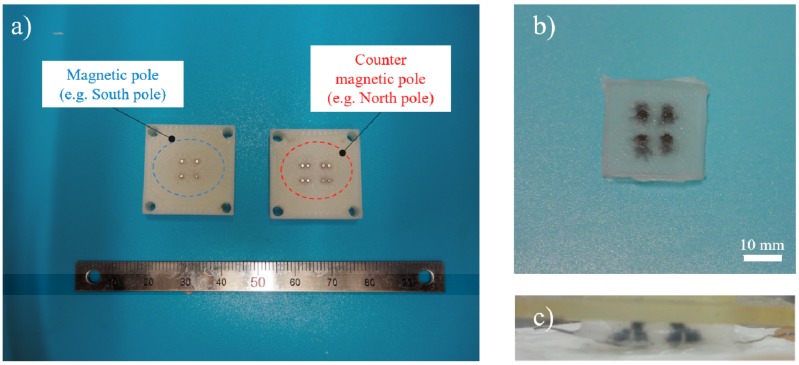
Arrayed soft-MPS: (**a**) mold for soft-MPS array; (**b**) picture of soft-MPS array (top view); (**c**) picture of soft-MPS array (side view).

The proposed sensor construction method is also effective for sensor arrays. A connection state can be achieved by utilizing the attraction between north and south poles. Moreover, a disconnection state can be achieved by utilizing the repulsion between two similar poles. It is also possible to arrange the sensor parts in an array by utilizing these properties (Figure 10). In the future, we will examine whether it is possible to detect force and position/displacement by such soft-MPS arrays.

## 5. Conclusions

In this study, we evaluated the manufacturing process of soft-MPSs, which are resistive tactile sensors, and their response characteristics. This sensor combines the high sensitivity of ferromagnetic powder with stability and durability by alignment along the magnetic field. Type-A soft-MPSs (i.e., with bottom electrodes) were fabricated and their characteristics were evaluated. It was confirmed that the soft-MPS (soft) (i.e., with low hardness) could detect a small force. The output voltage was evaluated for a pulsed input force, and a short time delay was observed between the input and output. In the future, modeling of soft-MPSs will be conducted to study the method of correcting for hysteresis, which is an inherent problem when using flexible materials, and distributed soft-MPSs will be developed by arraying and applied to the skin of robots. We believe that this design can be applied not only to soft materials but also to hard materials, in which tactile functions can be imparted to structural members such as resins.
